# Supplementary material for: Identification of Antitumor miR-30e-5p Controlled Genes; Diagnostic and Prognostic Biomarkers for Head and Neck Squamous Cell Carcinoma
Source: Genes (Basel). 2022 Jul 9;13(7):1225. doi: 10.3390/genes13071225 (PMC9322981; doi:10.3390/genes13071225)
Supplement: Supplementary file 1 [file genes-13-01225-s001.zip › genes-1791009-supplementary/Supplementary figures.pdf]

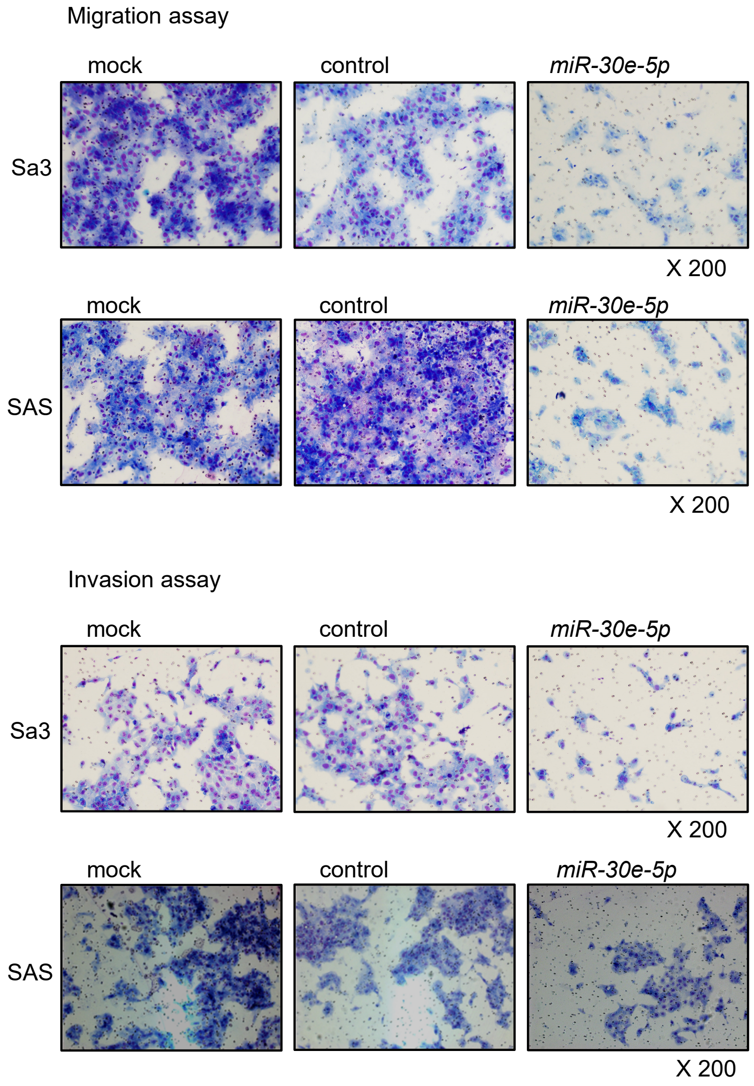

Figure S1: Photomicrographs of cells subjected to migration and invasion assays. Typical images of migration and invasion assays of Sa3 and SAS HNSCC cells following *miR-30e-5p* transfection.

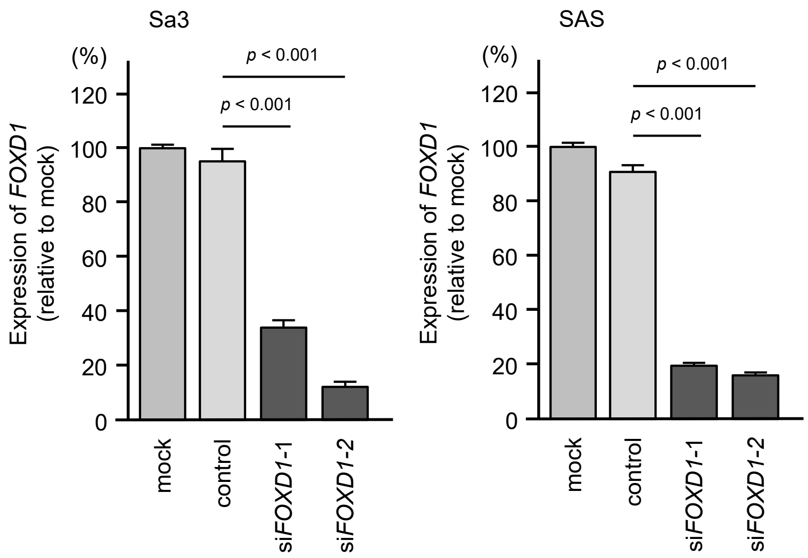

Figure S2: Efficiency of siRNA mediated *FOXD1* knockdown in HNSCC cell lines (Sa3 and SAS cells). The efficiency of *FOXD1* knockdown by two siRNAs targeting *FOXD1* (si*FOXD1*-1 and si*FOXD1*-2,) was evaluated by qRT-PCR. The *FOXD1* mRNA levels were measured at 72 h after siRNA transfection. *GAPDH* levels were used as internal controls.

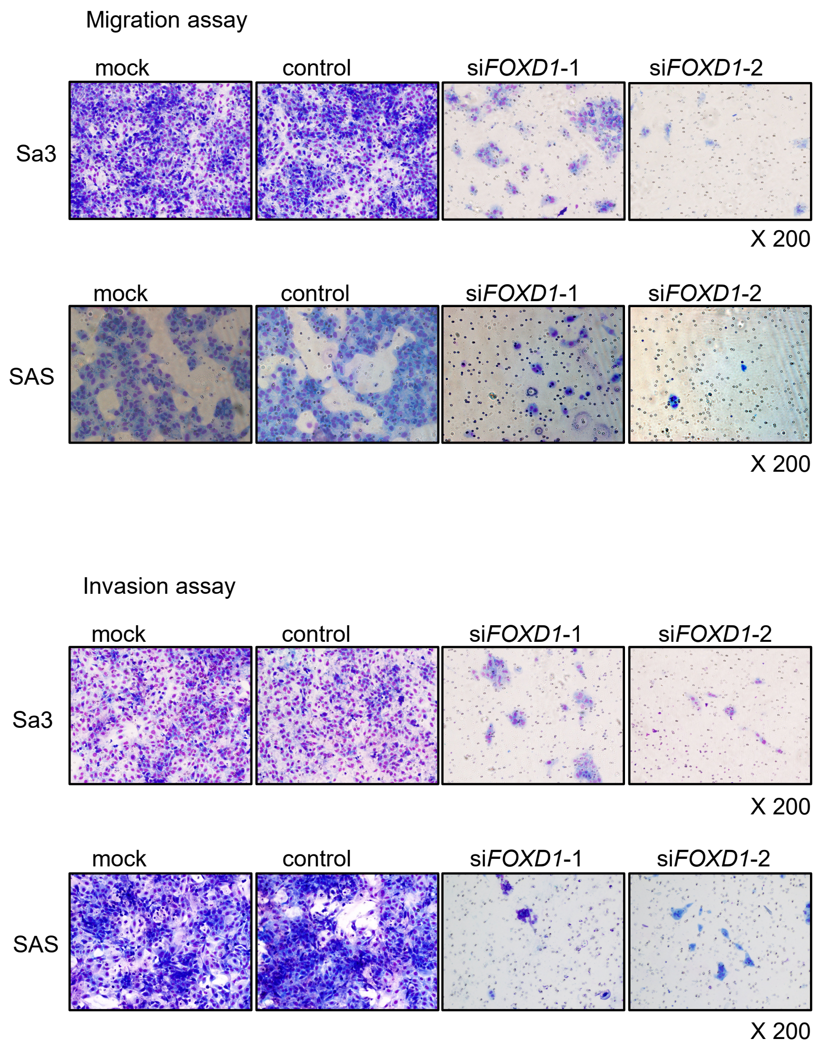

Figure S3: Photomicrographs of cells subjected to migration and invasion assays.
Typical images of migration and invasion assays of Sa3 and SAS HNSCC cells following si*FOXD1*-1 and si*FOXD1*-2 transfection.
